# Supplementary material for: Staufen2-mediated RNA recognition and localization requires combinatorial action of multiple domains
Source: Nat Commun. 2019 Apr 10;10:1659. doi: 10.1038/s41467-019-09655-3 (PMC6477676; doi:10.1038/s41467-019-09655-3)
Supplement: Supplementary file 3 — Reporting Summary [file 41467_2019_9655_MOESM3_ESM.pdf]

nature research

Corresponding author(s): Dierk Niessing

Last updated by author(s): Feb 7, 2019

Reporting Summary

Nature Research wishes to improve the reproducibility of the work that we publish. This form provides structure for consistency and transparency in reporting. For further information on Nature Research policies, see Authors & Referees and the Editorial Policy Checklist.

Statistics

For all statistical analyses, confirm that the following items are present in the figure legend, table legend, main text, or Methods section.

n/a	Confirmed	
☐	☒	The exact sample size (*n*) for each experimental group/condition, given as a discrete number and unit of measurement
☐	☒	A statement on whether measurements were taken from distinct samples or whether the same sample was measured repeatedly
☐	☒	The statistical test(s) used AND whether they are one- or two-sided<br>*Only common tests should be described solely by name; describe more complex techniques in the Methods section.*
☐	☒	A description of all covariates tested
☐	☒	A description of any assumptions or corrections, such as tests of normality and adjustment for multiple comparisons
☐	☒	A full description of the statistical parameters including central tendency (e.g. means) or other basic estimates (e.g. regression coefficient) AND variation (e.g. standard deviation) or associated estimates of uncertainty (e.g. confidence intervals)
☒	☐	For null hypothesis testing, the test statistic (e.g. *F*, *t*, *r*) with confidence intervals, effect sizes, degrees of freedom and *P* value noted<br>*Give P values as exact values whenever suitable.*
☒	☐	For Bayesian analysis, information on the choice of priors and Markov chain Monte Carlo settings
☒	☐	For hierarchical and complex designs, identification of the appropriate level for tests and full reporting of outcomes
☒	☐	Estimates of effect sizes (e.g. Cohen's *d*, Pearson's *r*), indicating how they were calculated

Our web collection on statistics for biologists contains articles on many of the points above.

Software and code

Policy information about availability of computer code

Data collection	Topspin 3.0 or Topspin 3.5; OmniSEC 5.12 software (Malvern); SpectraManager of the Jasco J-715 spectropolarimeter; Image Reader FLA-5000 series V1.0; FusionCapt Advance Solo4 software; Unicorn 5.2; Biacore 3000 control software 4.1; CCP4 version 7.0.068; MXCube v2 (ESRF); DA+ (SLS).
Data analysis	Origin Pro 9.0 (OriginLab) software; Topspin 3.0 or Topspin 3.5; Sparky 3; ccp4NMR v.2.4; RStudio v.1.1.456 with mixtools package and ggplot2 library; PyMol Version 1.7; CueMol Version 2.2.; OmniSEC 5.02 software (Malvern); BiaEvaluation software 4.1; CCP4 version 7.0.068, WinCoot version 0.8.6.1.; XDS version 20180808; SBGrid software bundle (2016); AutoRickshaw server (no version available); Fiji image analysis software version 1.0.

For manuscripts utilizing custom algorithms or software that are central to the research but not yet described in published literature, software must be made available to editors/reviewers. We strongly encourage code deposition in a community repository (e.g. GitHub). See the Nature Research guidelines for submitting code & software for further information.

Data

Policy information about availability of data

All manuscripts must include a data availability statement. This statement should provide the following information, where applicable:

- Accession codes, unique identifiers, or web links for publicly available datasets
- A list of figures that have associated raw data
- A description of any restrictions on data availability

Source data are available for Figures 1, 2, 4, 5, and 6. All structural data are available at the Protein Databank, https://www.rcsb.org, with accession number 6H0R. The data that support the findings of this study are available from the corresponding author upon reasonable request.

Field-specific reporting

Please select the one below that is the best fit for your research. If you are not sure, read the appropriate sections before making your selection.

☒ Life sciences ☐ Behavioural & social sciences ☐ Ecological, evolutionary & environmental sciences

For a reference copy of the document with all sections, see nature.com/documents/nr-reporting-summary-flat.pdf

Life sciences study design

All studies must disclose on these points even when the disclosure is negative.

Sample size	No predetermination of sample sizes were performed. Sample sizes were chosen based on existing standards in the field. For biochemical experiments at least 3 independent experiments were performed, for fly work between 10 and 37 samples were analyzed (details are described in Figure 6).
Data exclusions	No data were excluded, except for Biacore experiments where measurements with strong baseline drifts were excluded.
Replication	All experiments were repeated on different days and all replicates showed consistent results. In Biacore experiments in addition one value was measured in duplicates within one measurement.
Randomization	No randomization was performed because data collection was done by software algorithms, excluding human bias.
Blinding	Blinding was not necessary since data collection was done in an automated fashion.

Reporting for specific materials, systems and methods

We require information from authors about some types of materials, experimental systems and methods used in many studies. Here, indicate whether each material, system or method listed is relevant to your study. If you are not sure if a list item applies to your research, read the appropriate section before selecting a response.

Materials & experimental systems

n/a	Involved in the study	
☒	☐	Antibodies
☒	☐	Eukaryotic cell lines
☒	☐	Palaeontology
☐	☒	Animals and other organisms
☒	☐	Human research participants
☒	☐	Clinical data

Methods

n/a	Involved in the study	
☒	☐	ChIP-seq
☒	☐	Flow cytometry
☒	☐	MRI-based neuroimaging

Animals and other organisms

Policy information about studies involving animals; ARRIVE guidelines recommended for reporting animal research

Laboratory animals	Drosophila melanogaster, oocytes (i.e. females). Strains: stau[D3] (FBal0016165), stau[R9] (FBal0032815), αTub67C::GFPm6-Staufen (FBal0091177), oskar-Gal4 (FBtp0083699), w1118 (FBal0018186), y[1] M{vas-int.Dm}ZH-2A w[*]; PBac{y[+]-attP-3B}VK00033 (FBti0076453) females were used to facilitate psiC31-mediated insertion of UASp-rsEGFP2-mStau2 vectors.
Wild animals	Study did not involve wild-type animals
Field-collected samples	Study did not involve collection of field samples
Ethics oversight	No ethical approval or guidance was required for this study

Note that full information on the approval of the study protocol must also be provided in the manuscript.
